# HIV prevalence and associated risk factors among young tertiary student men who have sex with men (MSM) in Nairobi, Kenya: a respondent-driven sampling survey

**DOI:** 10.1186/s12981-023-00502-6

**Published:** 2023-02-06

**Authors:** Samuel Waweru Mwaniki, Peter Mwenda Kaberia, Peter Mwangi Mugo, Thesla Palanee-Phillips

**Affiliations:** 1 School of Clinical Medicine, Faculty of Health Sciences, University of the Witwatersrand, Johannesburg, South Africa; 2 University Health Services, University of Nairobi, State House Road, Nairobi, 00100 Kenya; 3 School of Mathematics, Faculty of Science and Technology, University of Nairobi, Nairobi, Kenya; 4 Kenya Medical Research Institute—Wellcome Trust Research Programme, Nairobi, Kenya; 5 Faculty of Health Sciences, Wits Reproductive Health and HIV Institute, University of the Witwatersrand, Johannesburg, South Africa; 6 Department of Epidemiology, School of Public Health, University of Washington, WA Seattle, USA

**Keywords:** College/university students, HIV prevalence, Respondent-driven sampling (RDS), Risk factors, Young men who have sex with men (YMSM)

## Abstract

**Background:**

Young men who have sex with men (MSM), are a key population at higher risk of HIV infection yet they are underrepresented in research. We conducted a bio-behavioral survey to estimate HIV prevalence and associated risk factors among tertiary student MSM (TSMSM) in Nairobi, Kenya.

**Methods:**

Between February and March 2021, 248 TSMSM aged ≥ 18 years who reported sex with another man in the past year participated in a respondent-driven sampling (RDS) based cross-sectional survey. Participants completed an electronically self-administered behavioral survey and provided a blood sample for HIV antibody testing, alongside urine, anorectal and oropharyngeal swabs for pooled testing of sexually transmitted infections using a multiplex nucleic acid amplification test. RDS-Analyst v.0.72 and Stata v.15 software were used for data analysis. Differences in proportions were examined using chi-square (χ^2^) test, and unweighted multivariate logistic regression was used to assess factors associated with HIV infection.

**Results:**

HIV prevalence among study participants was 8.3%, whereas the weighted prevalence was 3.6% (95% CI: 1.3–6.0%). Median ages of participants, and at self-reported first anal sex with a man were 21(interquartile range [IQR] 20-22) and 18 (IQR 17-19) years, respectively. A majority (89.3%) of TSMSM owned a smart phone, 46.5% had ever used a geosocial networking app for MSM such as Grindr ® to find a sex partner, and a third (33.6%) met their last sex partner online.  Almost three-quarters (71.3%) had > 1 male sex partner in the year before the survey. A third (34.3%) did not use condoms with their last sex partner, 21.2% received money from their last sex partner and 40.9% had taken alcohol/another drug during their last sexual encounter. HIV infection was associated with studying in private institutions (adjusted odds ratio[AOR] = 6.0; 95% confidence intervals [CI] : 1.2–30.0, p = 0.027), preferring a sex partner of any age—younger, same or older (AOR = 5.2; 95 CI: 1.1–25.2, p = 0.041), last sex partner being > 25 years (AOR = 6.4; 95% CI: 1.2–34.6, p = 0.030), meeting the last sex partner online (AOR = 4.2; 95% CI; 1.1–17.0, p = 0.043) and testing positive for *Neisseria gonorrhea* (AOR = 7.8; 95% CI: 2.0–29.9, p = 0.003).

**Conclusions:**

HIV prevalence among TSMSM in Nairobi is alarmingly high, demonstrating a need for tailored prevention and control interventions for this young key population.

## Introduction

The incidence of human immunodeficiency virus (HIV) has continued to fall globally [1]. Kenya has made laudable progress in the HIV response as shown by the sustained decline in HIV prevalence among adults (15–49 years) in the general population, from a high of ~ 10% in the mid-1990s to 4.5% in 2020 [2]. Whilst overall incidence has been declining, key populations such as men who have sex with men (MSM) still bear a disproportionate burden of the disease. Globally, despite MSM consisting only 1–3% of the adult male population, almost a quarter (23%) of all new HIV infections in 2020 were among MSM, with MSM having 25-fold greater odds of acquiring HIV than men in the general population [3]. In sub-Saharan Africa, the overall HIV prevalence is approximately 5 times higher among MSM than among men in the general population [4]. In Nairobi—Kenya’s capital, the prevalence of HIV among MSM is 25% [5] compared to 3.1% in the general male population (15–49 years old) [6], with 15.2% of all new HIV infections in the country occurring among MSM [7].

Kenya has a dedicated national program that leads the HIV response among key populations in the country [8]. Despite tremendous progress made in scaling up HIV prevention strategies among key populations, the program still grapples with challenges such as lack of updated bio-behavioral survey data and gaps in reaching adolescent and young key populations [9], including tertiary student MSM (TSMSM). In countries such as China where data is available, HIV prevalence among TSMSM is 4.1%, and despite TSMSM constituting only an estimated 8.5% of the total tertiary student population, they account for over 80% of all HIV infections in the tertiary institutions [10]. In South Africa, TSMSM comprise 6% of male tertiary students and have a HIV prevalence of 4.1%, which is greater than twice that of the general male tertiary student population—1.7% [11].

TSMSM like other young MSM (YMSM) may be involved in practices that heighten their risk of acquiring HIV. Upon joining tertiary institutions, they interact with peers who have similar same-sex behavior, experience more freedom from parental supervision, and have increased opportunities for socialization [12]. As a result, studies have shown that TSMSM seek casual sex partners online, have sex more often, and engage in condomless sex, group sex and sex work [10]. Additionally, TSMSM have been shown to have early sexual debuts, forced sex experiences, high sex partner turnover, concurrent sex partners, inconsistent condom use, and alcohol and drug use [13]. Taken together, these behaviors increase the risk of HIV infection for TSMSM. In many African countries, including Kenya, these behavioral risks are further amplified by structural factors such as criminalization of same-sex behavior [14], as well as societal stigma and discrimination towards individuals with same sex behavior [15].

Given the potential risk of HIV infection faced by TSMSM, it is important to understand what the HIV prevalence and associated risk factors are for this population. This information would be useful to stakeholders, including: policy makers, service providers, advocacy groups and TSMSM themselves, in informing tailored prevention and control responses for this population, and thereby contribute towards the ambitious goal of ending AIDS as a public health threat by 2030[16]. This study therefore estimated HIV prevalence and associated factors among TSMSM in Nairobi, Kenya.

## Methods

Study methods are detailed in the published study protocol [17], and summarized below:

### Study design and setting

This was a cross-sectional survey conducted between February and March 2021, just after COVID-19 restrictions were eased in Kenya and tertiary institutions allowed to re-open for in-person learning. Nairobi, the capital city of Kenya, was selected as the study area because of its large population of tertiary students spread across approximately 150 campuses of various universities and colleges [18], including University of Nairobi which is the largest university in the country [19].

### Study population

TSMSM were eligible to participate in the survey if they were: aged ≥ 18 years, provided proof of being a registered student in a university or college in Nairobi; were assigned male gender at birth, reported having had consensual receptive or insertive oral or anal sexual intercourse with another man in the last 12 months and provided written informed consent to participate in the study.

### Sample size calculation

The sample size for the survey was calculated to estimate the prevalence of HIV among TSMSM, based on World Health Organization’s (WHO) 2017 bio-behavioral survey guidelines for populations at higher risk of HIV infection [20]. A minimum sample size of 200 TSMSM was planned using the Cochran formula [21], with a design effect of 3 to account for clustering, estimated HIV prevalence of 4.1% based on a previous study among TSMSM in South Africa [11], a level of precision of 0.05 and 10% non-response.

### Sampling and recruitment process

Respondent-driven sampling (RDS) was used following findings from formative research that demonstrated the appropriateness and acceptability of the method for recruiting TSMSM into the survey [22]. In summary, RDS is a chain referral sampling method that integrates a mathematical model that weights the sample to compensate for the non-random sampling and minimizes old-style snowballing bias [23]. Six seeds were selected from the TSMSM who took part in the formative research. So as to reach varying networks of TSMSM, various characteristics were taken into consideration when selecting seeds, including: age, residence, sexual orientation identity, and preferred sexual role. Each seed and subsequent participants were issued with 3 coupons until the survey had recruited 120 participants (inclusive of seeds), then 2 coupons until the 150^th^ participant, after which no more coupons were issued. Each participant was reimbursed 1000 Kenyan shillings (equivalent to US$ 10) for their time and expenses related to transport to and from the study site, and 300 Kenyan shillings (equivalent to US$ 3) for every peer they recruited into the study.

### Data collection tools and procedures

The behavioral survey was conducted by self- administered interview on tablets using a questionnaire set up on REDCap® software (Vanderbilt University). The questionnaire was adapted for this study from a validated questionnaire available from the integrated HIV bio-behavioral surveillance toolbox [24]. The questionnaire contained questions on sociodemographic characteristics and sexual behavior, and was administered in English, the language of instruction in Kenyan tertiary institutions. The survey was conducted at a discreet study site located within the Nairobi central business district. The recruitment coupons had a map showing where the study site was located, and a telephone number which prospective participants could call for directions if they needed to.

### Biological specimen collection and testing

After completing the behavioral survey, participants were offered HIV testing based on the Kenyan national testing algorithm [25]. The testing algorithm is serial and recommends use of a screening test, followed by a confirmatory test if the screening test is positive. In cases where results of the confirmatory test disagree with those of the screening test, clients are referred to a laboratory where their samples are tested using an assay which is distinct from both the screening and confirmatory tests. Capillary blood from a finger prick was drawn by a trained HIV testing counselor. All samples were tested using the Determine® rapid test kit. Non-reactive tests were considered negative for HIV. Samples from those with reactive tests were subsequently tested with HIV-1/2 First Response® kit for confirmation of HIV seropositive status, except for participants who self-reported HIV seropositive status. There were no discordant results. Participants were offered pre and post HIV testing counselling. Participants who tested positive for HIV and were not on treatment were referred to facilities of their choice for HIV care and treatment. Participants who tested negative for HIV and were at high risk for HIV infection were referred for PrEP if not already initiated. Participants also provided urine, anorectal and oropharyngeal swabs for pooled *Chlamydia trachomatis* (CT), *Mycoplasma genitalium* (MG), *Neisseria gonorrhea* (NG) and *Trichomonas vaginalis* (TV) testing using a multiplex nucleic acid amplification test (Sacace Biotechnologies®, Italy). HIV testing was done at the study site whereas testing for the other sexually transmitted infections (STIs) was done in a licensed and accredited commercial laboratory within the Nairobi metropolis. All participants were offered condoms and water-based lubricants.

### Ethical considerations

The study was approved by University of the Witwatersrand Human Research Ethics Committee-Medical and University of Nairobi-Kenyatta National Hospital Ethics and Research Committee. All participants provided written informed consent. The following measures were taken to protect study participants: no identifying information was written on study documents nor laboratory specimens associated with a participant; all paper-based study materials were stored in locked file cabinets, in locked offices and access granted to only authorized study staff; electronic data was stored in a password-protected computer accessible only to authorized study staff; and same-sex sexual behavior was not reported to the authorities. All staff working with participants were trained on the importance of maintaining confidentiality, and signed confidentiality agreements prior to involvement in the study. The study also received a letter of support from the Kenyan Ministry of Health through the National AIDS and STI Control Program (NASCOP).

### Data analysis

Data analysis excluded 6 seeds who were purposely selected to begin the RDS recruitment. The weighted HIV prevalence rate with 95% confidence intervals (CI) was calculated using RDS Analyst (RDS-A) software version 0.72 [26], where Gile’s successive sampler (SS) estimator was used for weighting. Gile’s SS estimator takes into consideration the self-reported network sizes of participants, recruitment patterns and estimated size of the study population [27]. As per recommendation [28], an estimated population size of 8406 for TSMSM (1.45% of male tertiary students in the Nairobi metropolis) was used, with 0.95 CI and 1000 bootstraps.

Stata version 15 (College Station, TX: StataCorp LLC) was used for further analysis of the unweighted data. Some continuous variables such as age and self-reported number of sex partners were converted into binary categories. Categorical variables were summarized using proportions, and differences in proportions examined using chi-square (χ^2^) test. Bivariate analysis and multivariate logistic regression models were used to measure associations between various factors and HIV infection. All exposure variables with a p ≤ 0.2 in bivariate analysis were included in the  final multivariate model. Other variables with p > 0.2 but considered to be confounders were included in the multivariate model. Unweighted regression models were used since they have been shown to perform better than weighted regression models for RDS data [29].

## Results

### Sociodemographic characteristics of TSMSM

**Fig. 1:**
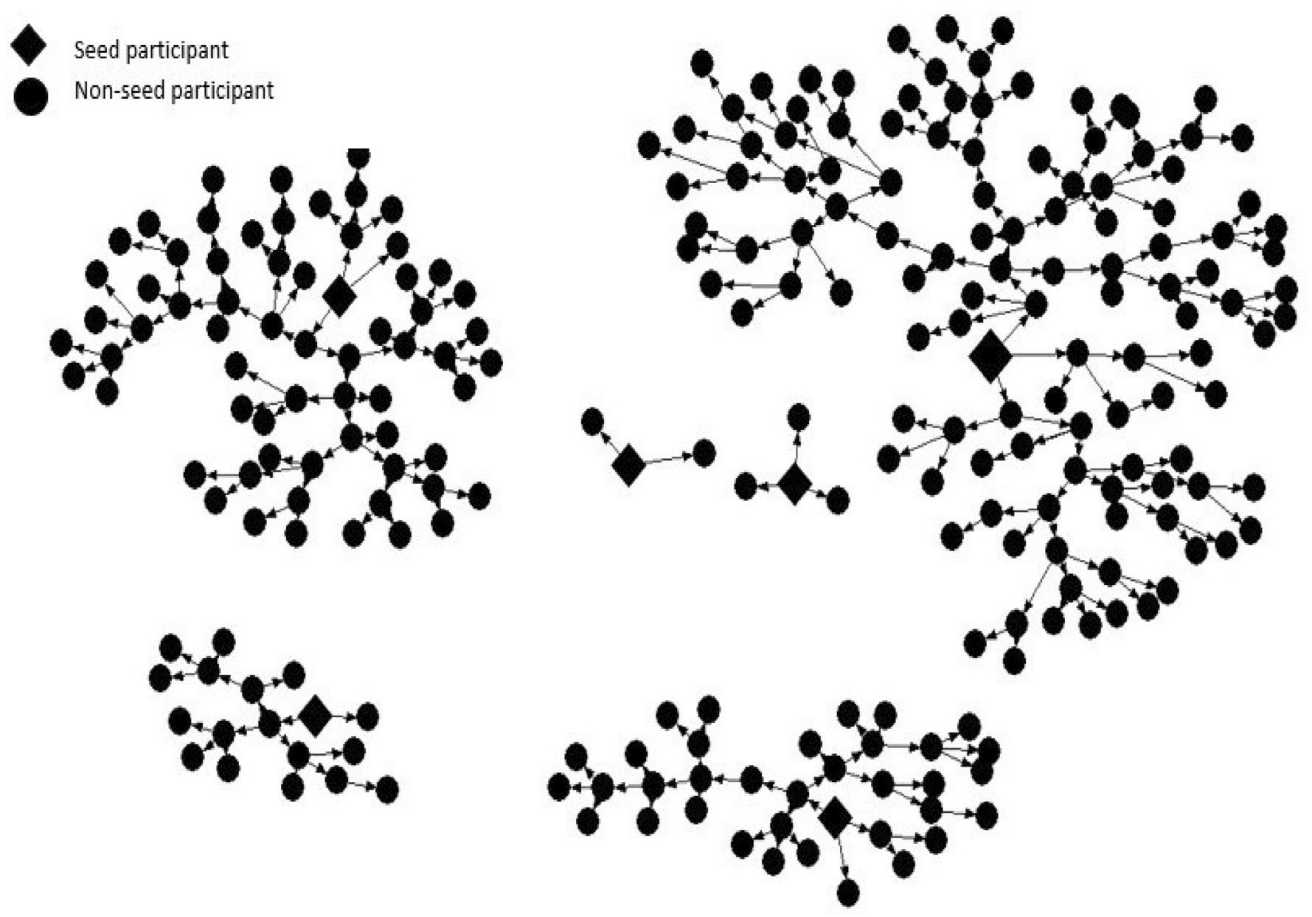
Recruitment of tertiary student men who have sex with men in Nairobi (*n* = 248)

A total of 248 TSMSM were recruited including 6 seeds. The final sample had a minimum of one and maximum of eight recruitment waves, as depicted by the recruitment networks in Fig. 1. The median age of participants was 21 years (IQR 20–22), with 96.3% ≤ 24 years, and the oldest being 30 years old (Table 1). A majority (58.3%) were university students, almost three-quarters (71.9%) were in public institutions and four-fifths (79.3%) resided in college or rented hostels outside their campuses. A majority (89.3%) owned a smart phone at the time of the study.

**Table 1:** Characteristics of TSMSM (total and by HIV status) in Nairobi, Kenya (*n* = 242)

Variable	Category	n (%)	HIV negative n (%)	HIV positive n (%)	*p* value
Sociodemographic characteristics					
Age (years)	18–20	89 (36.8)	83 (93.3)	6 (6.7)	0.512
	21–30	153 (63.2)	139 (90.8)	14 (9.2)	
Level of institution	University	141 (58.3)	131 (93.0)	10 (7.0)	0.434
	College	101 (41.7)	91 (90.1)	10 (9.9)	
Type of institution	Public	174 (71.9)	165 (94.8)	9 (5.2)	0.005*
	Private	68 (28.1)	57 (83.8)	11 (16.2)	
Type of course	Engineering and technology	103 (42.6)	97 (94.2)	6 (5.8)	0.551
	Health, natural and social sciences	53 (21.9)	48 (90.6)	5 (9.4)	
	Business management	48 (19.8)	44 (91.7)	4 (8.3)	
	Arts and humanities	38 (15.7)	33 (86.8)	5 (13.2)	
Year of study^a^	1st and 2nd year	153 (66.0)	140 (91.5)	13 (8.5)	0.812
	3rd and 4th year	79 (34)	73 (92.4)	6 (7.6)	
Residence	College/rented hostel	192 (79.3)	180 (93.8)	12 (6.2)	0.026*
	With family	50 (20.7)	42 (84.0)	8 (16.0)	
Ever attended boarding school	Yes	203 (16.1)	188 (92.6)	15 (7.4)	0.259
	No	39 (83.9)	34 (87.2)	5 (12.8)	
Main source of finances	Parents/guardians	137 (56.6)	127 (92.7)	10 (7.3)	0.692
	Employment	65 (26.9)	58 (89.2)	7 (10.8)	
	Bursary/scholarship	40 (16.5)	37 (92.5)	3 (7.5)	
Had smartphone at time of the study	Yes	216 (89.3)	196 (90.7)	20 (9.3)	0.105
	No	26 (10.7)	26 (100.0)	0 (0.0)	
Sexual orientation	Gay	153 (63.2)	140 (91.5)	13 (8.5)	0.655
	Bisexual	80 (33.1)	73 (91.3)	7 (8.7)	
	Heterosexual	9 (3.7)	9 (100.0)	0 (0.0)	
Gender identity	Cisgender male	219 (90.5)	201 (91.8)	18 (8.2)	0.937
	Transgender female	23 (9.5)	21 (91.3)	2 (8.7)	
Sexual behavior characteristics					
Ever had vaginal sex with a woman	Yes	130 (53.7)	123 (94.6)	7 (5.4)	0.080
	No	112 (46.3)	99 (88.4)	13 (11.6)	
Age at first anal sex with a man	≤ 18 years	137 (57.0)	124 (90.5)	13 (9.5)	0.455
	> 18 years	103 (43.0)	96 (93.2)	7 (6.8)	
Preferred age of sex partner	Same/younger age	187 (77.6)	178 (95.2)	9 (4.8)	0.001*
	Any age	36 (15.0)	29 (80.6)	7 (19.4)	
	Older	18 (7.4)	14 (77.8)	4 (22.2)	
Age of last sexual partner (years)	15–24	204 (84.7)	193 (94.6)	11 (5.4)	< 0.001*
	≥ 25	37 (15.3)	28 (75.7)	9 (24.3)	
Sexual role at first anal sex with a man	Insertive	103 (42.8)	95 (92.2)	8 (7.8)	0.514
	Receptive	83 (34.4)	74 (89.2)	9 (10.8)	
	Versatile	55 (22.8)	52 (94.6)	3 (5.4)	
Preferred sexual role	Insertive	117 (48.6)	109 (93.2)	8 (6.8)	0.512
	Versatile	75 (31.1)	69 (92.0)	6 (8.0)	
	Receptive	49 (20.3)	43 (87.8)	6 (12.2)	
Nature of first anal sex with a man	Consensual	201 (83.4)	182 (90.6)	19 (9.4)	0.145
	Coerced/forced	40 (16.6)	39(97.5)	1 (2.5)	
Number of men had anal sex within last 12 months	One (1)	69 (28.7)	68 (98.6)	1 (1.4)	0.015*
	More than one (1)	172 (71.3)	153 (89)	19 (11.0)	
Participated in group sex within last 12 months	No	202 (83.8)	186 (92.0)	16 (8.0)	0.628
	Yes	39 (16.2)	35 (89.7)	4 (10.3)	
Ever used a geosocial networking app for MSM to find a sex partner	Yes	112 (46.5)	95 (84.8)	17 (15.2)	< 0.001*
	No	129 (53.6)	126 (97.7)	3 (2.3)	
Where last sex partner was met	Offline	160 (66.4)	153 (95.6)	7 (4.4)	0.002*
	Online	81 (33.6)	68 (84.0)	13 (16.0)	
Used condom with last sexual partner	Yes	159 (65.7)	145 (91.2)	14 (8.8)	0.673
	No	83 (34.3)	77 (92.8)	6 (7.2)	
Received money from last sex partner	Yes	51 (21.2)	47 (92,2)	4 (7.8)	0.894
	No	190 (78.8)	174 (91.6)	16 (8.4)	
Taken alcohol/other drug during last sexual encounter	Yes	99 (40.9)	89 (89.9)	10 (10.1)	0.388
	No	143 (59.1)	133 (93.0)	10 (7.0)	
Sexually transmitted infections test results					
* Chlamydia trachomatis*	Negative	100 (41.3)	94 (94.0)	6 (6.0)	0.283
	Positive	142 (58.7)	128 (90.1)	14 (9.9)	
* Mycoplasma genitalium*	Negative	217 (89.7)	202 (93.1)	15 (6.9)	0.024*
	Positive	25 (10.3)	20 (80.0)	5 (20.0)	
* Neisseria gonorrhea*	Negative	206 (85.1)	194 (94.2)	12 (5.8)	0.001*
	Positive	36 (14.9)	28 (77.8)	8 (22.2)	
* Trichomonas vaginalis*	Negative	239 (98.8)	219 (91.6)	20 (8.4)	0.601
	Positive	3 (1.2)	3 (100.0)	0 (0.0)	

*TSMSM* tertiary student men who have sex with men

^*^Significant difference in proportions

^a^Ten (10) missing values not included in the analysis

Recruitment was initiated by six seeds who were issued with 418 coupons, 244 coupons were returned and out of these, 242 met the eligibility criteria for study participation and were subsequently enrolled into the study. The waves of recruitment per seed ranged from 1 to 8. Diamonds and circles indicate seed and non-seed participants, respectively.

### Sexual behavior characteristics of TSMSM

The median age at reported anal sexual debut with a man was 18 years (IQR 17–19) and most of the sexual debuts (83.4%) were consensual, as shown in Table 1. Almost three-quarters (71.3%) had more than one sex partner, with 16.2% having participated in group sex within the last year. Approximately half (46.5%) had ever used a geosocial networking app for MSM such as Grindr ® to find a sex partner, with one-third (33.6%) having met their last sex partner online. Approximately two-thirds (65.7%) and more than three-quarters (78.1%) had used condoms and lubricants, respectively, with their last sex partner. One-fifth (21.2%) received money from their last sex partner and less than half (40.9%) had taken alcohol or another drug during their last sexual encounter.

### HIV prevalence, differences in proportions, and associated factors

The HIV prevalence among the study participants was 8.3%. The weighted HIV prevalence for the study population (TSMSM in Nairobi) was 3.6% (95% CI: 1.3%—6.0%). Three-quarters (77.3%) of those who tested positive for HIV knew of their HIV serostatus prior to testing in the study and all of these participants reported being on antiretroviral therapy. As illustrated in Table 1, HIV prevalence was significantly higher among TSMSM studying in private institutions (16.2% vs 5.2%, p = 0.005), residing at home with their families (16.0% vs 6.2%, p = 0.026), who preferred older sex partners (22.2% vs 4.8%, p = 0.001), whose last sex partner was > 25 years old (24.3% vs 5.4%, p < 0.001), who had more than one sex partner in the last year (11.0% vs 1.4%, p = 0.015), who had ever used a geosocial networking app for MSM (15.2% vs 2.3%, p < 0.001), who met their last sex partner online (16.0% vs 4.4%, p = 0.002) and those who tested positive for MG (20.0% vs 6.9%, p = 0.024) and NG (22.2% vs 5.8%, p = 0.001).

The results of bivariate and multivariate logistic regression are presented in Table 2. In multivariate analysis, independent risk factors for HIV infection were: studying in private institutions (Adjusted Odds Ratio [AOR] = 6.0; 95% CI: 1.2–29.9, p = 0.027), preferring a sex partner of any age (AOR = 5.1, 95% CI: 1.1–25.2, p = 0.041), having a last sex partner > 25 years old (AOR = 6.4; 95% CI: 1.2–34.6, p = 0.030), meeting the last sex partner online (AOR = 4.2; 95% CI; 1.1–17.0, p = 0.043) and testing positive for NG (AOR = 7.8, 95% CI: 2.0–29.9, p = 0.003).

**Table 2:** Unweighted logistic regression of HIV infection and various factors among TSMSM in Nairobi, Kenya (*n*= 239)

Variable	Category	Unadjusted Odds Ratio (95% CI)	*p* value	Adjusted Odds Ratio (95% CI)	*p* value
Sociodemographic characteristics					
Type of institution	Public	1			Ref
	Private	3.5 (1.4–9.0)	0.008	6.0 (1.2–30.0)	0.027*
Residence	College/rented hostel	1			Ref
	With family	2.9 (1.1–7.4)	0.031	3.0 (0.7–13.2)	0.572
Sexual behavior characteristics					
Age at first anal sex with a man	≤ 18 years	1			Ref
	> 18 years	0.7 (0.3–1.8)	0.457	0.6 (0.2–2.4)	0.516
Preferred age of sex partner	Same/younger age	1			Ref
	Any age	4.8 (1.6–13.8)	0.004	5.2 (1.1–25.2)	0.041*
	Older	5.7 (1.5–20.7)	0.009	1.1 (0.1–9.2)	0.948
Age of last sexual partner	15–24	1			Ref
	≥ 25	5.6 (2.1–14.8)	< 0.001	6.4 (1.2–34.6)	0.030*
Sexual role at first anal sex with a man	Versatile	1			Ref
	Insertive	1.4 (0.4–5.7)	0.588	4.9 (0.7–32.7)	0.097
	Receptive	2.1 (0.5–8.2)	0.280	2.0 (0.3–12.7)	0.476
Preferred sexual role	Insertive	1			
	Versatile	1.2 (0.4–3.6)	0.763	0.5 (0.1–2.4)	0.380
	Receptive	1.9 (0.6–5.8)	0.259	1.6 (0.3–8.6)	0.572
Nature of first anal sex with a man	Coerced/forced	1			Ref
	Consensual	4.1 (0.5–31.3)	0.177	0.1 (0.1–1.5)	0.090
Number of men had anal sex with in last 12 months	One	1			Ref
	More than one	8.4 (1.1–64.4)	0.040	8.3 (0.7–94.6)	0.088
Participated in group sex within last 12 months	No	1			Ref
	Yes	1.3 (0.4–4.2)	0.629	0.9 (0.2–4.3)	0.877
Where last sex partner was met	Offline	1			Ref
	Online	4.2 (1.6–11.0)	0.004	4.2 (1.1–17.0)	0.043*
Used condom with last sex partner	No	1			Ref
	Yes	1.2 (0.5–3.4)	0.673	4.3 (0.9–20.3)	0.065
Received money from last sex partner	Yes	1			Ref
	No	1.1 (0.3–3.4)	0.894	3.5 (0.7–18.7)	0.139
Taken alcohol/other drug during last sexual encounter	No	1			Ref
	Yes	1.5 (0.6–3.7)	0.390	2.3 (0.6–8.7)	0.214
Sexually transmitted infections test results					
* Mycoplasma genitalium* test	Negative	1			Ref
	Positive	3.3 (1.1–10.2)	< 0.001	2.1 (0.5–9.1)	0.293
* Neisseria gonorrhea* test	Negative	1			
	Positive	4.6 (1.7–12.3)	< 0.001	7.8 (2.0–29.9)	0.003*

TSMSM: tertiary student men who have sex with men

Three records had missing values and were excluded from the logistic regression

*Independent risk factor for HIV infection

## Discussion

Our study found an estimated HIV prevalence of 8.3% among study participants and a RDS-weighted HIV prevalence of 3.6% (95% CI: 1.3–6.0%) among TSMSM in Nairobi. The weighted HIV prevalence observed among TSMSM in our study (96% aged 18–24 years) was six times higher than that of young Kenyan men in the general population aged 18–24 years, which is 0.6%[6]. Accordingly, the recent call to have programming that caters for the HIV prevention needs of YMSM in Africa [30], is quite relevant for TSMSM in Nairobi. The weighted HIV prevalence observed is comparable to the 4.1% HIV prevalence found among TSMSM in both China [10] and South Africa [11]. Notably, with only 77.3% of study participants knowing their HIV status to be seropositive before the study, there is need to scale up HIV testing in this population so as to reach the first 95 of the UNAIDS fast-track 95-95-95 [31]. HIV self-testing is readily available and acceptable in Kenya [32], and should thus be harnessed to increase testing rates in this key population.

Higher HIV prevalence was observed among TSMSM studying in private institutions and staying at home with their families. We hypothesize that most TSMSM who study in private institutions and stay with their families have predominantly lived most of their lives in the city, which is more liberal than the rural areas of the country. Therefore, it is likely that these TSMSM could have started experimenting with anal sex at an earlier age than their rural counterparts who due to costs, mostly tend to join the public institutions and stay either in campus or rented accommodation outside their campuses. For these reasons, there could be an increased exposure period for the former, leading to the elevated risk of HIV infection. Indeed, even higher HIV prevalence (30.3%) than we found, has been observed among YMSM in urban Indonesia [33], a country where it is not illegal to be homosexual.

Meeting sex partners online was significantly associated with HIV infection. This finding is consistent with that of a previous study in Kenya which showed that MSM who sought sex partners online had four-fold greater odds of having HIV infection compared to those who sought sex partners from physical sites [34]. Additional research in sub-Saharan Africa has demonstrated that MSM who seek sex partners online have higher prevalent HIV infection and are more likely to be younger [35], like our study participants. Young MSM, including TSMSM who use the internet to find sex partners are a critical population to target for HIV interventions, since they are inclined to have increased levels of sexual risk behavior, and the internet itself may be a promising mechanism to deliver interventions [36]. Like in our study where 89.6% owned a smart phone, another study showed that 95.6% of tertiary students in Kenya own smart phones [37], and use the phones to access the internet. Future YMSM-focused risk reduction interventions should leverage the internet to host campaigns and safe sex messaging efforts to directly and discretely reach TSMSM who predominantly seek sex partners online. This is necessary since TSMSM—as a result of criminalization of homosexuality, societal stigma and discrimination, may be afraid to seek services from programs that largely depend on physical outreach, thus resulting in missed opportunities for HIV prevention.

Another significant risk factor identified for HIV infection was having reported the last sex partner being > 25 years. This is consistent with the findings of a study in the USA which showed that among YMSM, the odds of HIV infection were significantly elevated as the age of sexual partners increased [38]. This could be explained by the fact that risk of HIV infection generally increases with age, thus increasing the risk of HIV infection among YMSM who have sex with older male partners. In addition, practices that increase the risk of HIV infection such as condomless anal sex have been shown to be common in age-disparate sexual relationships among MSM, and are significantly associated with unrecognized HIV infection [39]. Previous research has shown that YMSM are motivated to seek older MSM partners for economic and social support [40]. Subsequently, the economic power imbalance between YMSM and older MSM may diminish the former’s ability to negotiate for safer sex [41], with YMSM previously reporting sexual coercion by older MSM [42]. Since the YMSM—older MSM partnerships are more likely to persist than wane, HIV prevention programs for YMSM should include information about the HIV risk associated with older MSM partners and skills for negotiating for safer sex, particularly the correct and consistent use of condoms. Additionally, TSMSM should be provided with more tools for HIV prevention, including oral pre-exposure prophylaxis (PrEP) which is available in Kenya [43], and offers YMSM more control on usage as compared to condoms which need negotiation between sex partners. As well, since YMSM may choose more pleasurable (condomless sex) over safer sex, PrEP would offer them the opportunity to enjoy sex without fear of HIV infection, the risk of acquiring other STIs notwithstanding [44].

A notable independent risk factor for HIV infection that was observed in our study was testing positive for NG. This coupled with the high prevalence of CT observed (58.7%) has implications for HIV control and prevention. The causal relationship between prevalent CT/NG and incident HIV has previously been established, with CT/NG shown to increase both the risk of HIV acquisition and transmission [45]. Among MSM, rectal CT/NG re-infection has been associated with increased risk of HIV seroconversion [46], with modelling studies demonstrating that 10.4% of incident HIV infections are attributable to CT/NG infection [47]. TSMSM should therefore be encouraged to have diagnostic STI screening often, depending on sexual activity and risk-taking, and thereafter receive appropriate care. This would help mitigate the deleterious synergy between STI and HIV, as well as forestall morbidities such as infertility that occur secondary to some STI.

Although not independently associated with HIV infection in our study, other behaviors that increase the risk of HIV infection were common. These include engaging in condomless sex, group sex, transactional sex, alcohol and drug use during sex, as well as having multiple sex partners. These findings are consistent with what has been observed among TSMSM in China [10] and South Africa [13]. Behavioral change is therefore urgently needed in this population to augment the biomedical tools available for HIV prevention.

To our knowledge, this study was the first one to assess the prevalence and associated factors of HIV among TSMSM in Kenya, and possibly in sub-Saharan Africa using the RDS method. These findings should be viewed in light of some limitations. Firstly, RDS is not a classical probabilistic sampling method. To offset this limitation, when calculating our sample size, we applied a design effect of 3 to account for the clustering that occurs due to homophily and minimize the traditional bias associated with snowball sampling. We also excluded from analysis the 6 seeds who were purposely selected to start off recruitment. Secondly, since we asked about what happened in the past year, there was a possibility of recall bias. To mitigate this, we aimed to include in our questionnaire either single or multiple choice questions and have very few instances where participants needed to type out answers. We also limited the number of questions about events that happened more than 12 months before the study. Thirdly, we asked questions about sexual behavior which may be affected by social desirability bias. To minimize this, participants self-administered the survey on an online platform accessed through tablet computers, since this has been shown to be more effective in offsetting this kind of bias, as compared to face-to-face interviews [48]. Fourthly, the cross-sectional nature of the study limits causal inference of the independent risk factors identified for HIV infection. Finally, though we used a comprehensive set of variables in the logistic regression analysis, we cannot rule out the possibility of residual confounding from other variables such as experiences of stigma, discrimination and violence. Despite these limitations, this survey offers valuable lessons on the burden of HIV and associated factors among TSMSM, and hopefully portends the development of tailored interventions for the HIV response in this key yet understudied population.

## Conclusions

In summary, HIV prevalence among TSMSM in Nairobi is 6 times higher than that of Kenyan young men of comparable age. As a result, urgent efforts to develop bespoke HIV prevention and control interventions for this population are called for. Such interventions should seek to enhance HIV testing (including through HIV self-testing), as well as promote STI screening and treatment. TSMSM should be provided with education messages on the attendant risk of HIV infection in age-disparate relationships, and empowered to practice safer sex through use of both condoms and PrEP. Further, there is need for collaboration between tertiary institutions and relevant agencies of the ministry of health such as National AIDS and STI Control Program (NASCOP), in providing HIV prevention and control services to TSMSM so as to avert new HIV infections in this population. Given its widespread availability and use in Kenya, the internet is an innovative tool that should be harnessed for delivery of these interventions.

## Data Availability

The datasets used and/or analyzed during this study is not publicly available, but may be available from the corresponding author upon reasonable request, and with permission from University of the Witwatersrand Human Research Ethics Committee-Medical and University of Nairobi-Kenyatta National Hospital Ethics and Research Committee.
